# Skin Cancer, Irradiation, and Sunspots: The Solar Cycle Effect

**DOI:** 10.1155/2014/538574

**Published:** 2014-07-14

**Authors:** Edward Valachovic, Igor Zurbenko

**Affiliations:** School of Public Health, State University of New York at Albany, Rensselaer, NY 12144, USA

## Abstract

Skin cancer is diagnosed in more than 2 million individuals annually in the United States. It is strongly associated with ultraviolet exposure, with melanoma risk doubling after five or more sunburns. Solar activity, characterized by features such as irradiance and sunspots, undergoes an 11-year solar cycle. This fingerprint frequency accounts for relatively small variation on Earth when compared to other uncorrelated time scales such as daily and seasonal cycles. Kolmogorov-Zurbenko filters, applied to the solar cycle and skin cancer data, separate the components of different time scales to detect weaker long term signals and investigate the relationships between long term trends. Analyses of crosscorrelations reveal epidemiologically consistent latencies between variables which can then be used for regression analysis to calculate a coefficient of influence. This method reveals that strong numerical associations, with correlations >0.5, exist between these small but distinct long term trends in the solar cycle and skin cancer. This improves modeling skin cancer trends on long time scales despite the stronger variation in other time scales and the destructive presence of noise.

## 1. Introduction

### 1.1. Skin Cancer

Cancer is known to have genetic and environmental risk factors. Particular types of cancer can have a greater association with one factor than with others. One such example is that of skin cancer. Skin cancer (SC) is an unregulated growth of abnormal skin cells named after the type of skin cell from which they arise, for example, basal, squamous, and melanoma. Skin cancer is the most common cancer in the United States, affecting over 2 million annually [1]. Melanoma risk doubles with a history of more than five sunburns [2]. Risk, likewise, doubles after just one blistering sunburn during childhood [3].

The relationship between sunlight exposure, in particular that of the ultraviolet portion of the electromagnetic spectrum, and the increased likelihood of developing skin cancer has been a frequent subject of research. Studies indicate that approximately 90% of nonmelanoma skin cancer is associated with ultraviolet exposure [4] while this value is approximately 86% for melanoma skin cancer [5]. The pronounced seasonal component in the diagnosis of skin cancer is a frequent subject of research in order to examine the effect of UV exposure [6]. Often overlooked in this relationship are the nature and the characteristics of the sunlight. Solar intensity is the subject of little epidemiological research and it is largely treated as invariant. However, solar irradiation carries a unique fingerprint that when properly identified enables a new examination of this relationship.

### 1.2. The Solar Cycle

The sun is an engine of nuclear fusion and as a result exhibits several measurable characteristics associated with solar nuclear activity. One defining feature is that solar activity is not constant across time. The intensity of solar activity undergoes an approximate 11-year cycle resulting in a naturally occurring pattern of maximums and minimums. Likewise, many characteristics associated with solar activity exhibit a strong cyclic nature with this approximate 11-year period [7]. This phenomenon is well documented and studied across various academic fields, such as astronomy, physics, and the atmospheric and environmental sciences [8].

Some of the solar characteristics that exhibit the solar cycle include electromagnetic radiation, irradiation, luminosity, magnetic field strength, magnetic polarity, flares, sunspot number, and solar wind. Every characteristic variable has unique methods of measurement and measurement histories. Likewise, the relationship of each variable to the underlying solar cycle phenomenon is different and not automatically synchronous or perfectly correlated [9]. Though most variables exhibit their own 11-year cycle, each one may have its own amplitude and phase shift [10]. This makes each variable more or less suitable for the particular field or application of interest. In this study electromagnetic radiation, ultraviolet radiation, in particular, is of clear interests and preference due to its known association with skin cancer risk.

Total solar irradiance (TSI) is the measure of the sum total power across the entire electromagnetic spectrum emitted by the sun and received per unit surface area. Measurements may be made in orbit (OTSI) or at ground elevations (GTSI). Another solar characteristic, sunspots, is patches on the surface of the sun characterized by locally diminished brightness and temperature and corresponds to the changing magnetic field within the sun [11]. Like TSI and the other solar characteristics, sunspot number (SN) varies cyclically approximately in phase with TSI sharing an 11-year period [12]. Therefore it is highly correlated with TSI but on the surface of the Earth TSI is affected by other factors such as latitude. It will be best to measure the direct effect of TSI on skin cancer when more data is available. However, in the absence of sufficient data, we rely upon the highly correlated sunspot number with a far longer history of observation and consistent historical records beginning in the early 18th century [7].

### 1.3. Separation of Time Scales

These time series data sets illustrate one difficulty when investigating long term trends and correlations between variables in order to determine association structures and causative relationships. The relative strength of the components from one time scale can obscure those operating in a different time scale, interfering with the detection of a signal of interest. The separation of different time scales into constituent components allows for the proper unobstructed analysis. Kolmogorov-Zurbenko (KZ) filters (http://en.wikipedia.org/wiki/Kolmogorov%E2%80%93Zurbenko_filter/) are well suited to this task of separating and screening interfering time scales for signal detection. Analysis is further complicated by skin cancer, unlike many cancers, not having extensive periods of time between initiation, onset, and detection [13]. It has been shown that a history of severe sun burns is a risk factor for skin cancer later in life. These are events that may have occurred decades earlier. Direct comparison of data without accounting for possible disease latencies may produce inconclusive results or erroneous effects.

There is scarce research into global effects of solar irradiation and intensity changes upon individual diseases and disease rates. The objective of this study is to examine the long term changes in SC and SN as a proxy for TSI and investigate the solar cycle and skin cancer relationship. This study demonstrates KZ filtration of signals into different time scale components precisely because different time scales result from different sources and may interfere in the analysis of each individual component [14]. Crosscorrelations at all reasonable latencies are calculated to identify the latencies of peak correlation between time series datasets. These peak correlations and respective latencies enable regression analysis in an attempt to model the relationship and identify a coefficient of influence.

## 2. Methods

### 2.1. Data Sources

The sunspot number time series dataset comes from a record with dates spanning the years from 1749 until the present consisting of monthly observed sunspot counts. This record is available from the Solar Influences Data Analysis Center (http://www.sidc.oma.be/). Orbital measurements of TSI were recorded from the ACRIM, or Active Cavity Radiometer Irradiance Monitor, series of satellite instruments in the years between 1974 and 2006. As a measure of power per unit surface area they are recorded as watts per meter squared (http://www.acrim.com/). The ACRIM composite is a TSI data series that is primarily composed of these instrument readings [15]. Approximately 10 percent of the data of the series is missing, the longest span being called the ACRIM gap between ACRIM-1 and ACRIM-2, and is filled and scaled with data from Earth Radiation Budget (ERB) experiments, Nimbus7/ERB, to relate ACRIM-1 and ACRIM-2 results. There are other composite approaches that use different subsets of satellite TSI data, ACRIM gap ratios, and different modelling, but the ACRIM composite suits this analysis. Ground observations of total solar irradiation, GTSI, for this study were recorded in New Mexico. GTSI spans the years 1961–2010 and originated from the National Solar Radiation Data Base (rredc.nrel.gov). These are statistical summaries of solar data originally recorded hourly, compiled as averages of daily total solar energy for each given month. Hempelmann and Weber explore the strong correlations between SN and TSI surface level irradiance [16].

Skin cancer records arise from case level data in the SEER, or Surveillance, Epidemiology, and End Results database, 1973–2009 (Surveillance, Epidemiology, and End Results (SEER) Program (http://www.seer.cancer.gov/) Research Data (1973–2009), National Cancer Institute, DCCPS, Surveillance Research Program, Surveillance Systems Branch, released April 2012, based on the November 2011 submission). The SEER sites included for this study are the states of Connecticut, Hawaii, New Mexico, Utah, and Iowa and the cities of Oakland and Detroit throughout the years 1984–2009. As retrospective observational records these sites were not purposefully selected but they rather represent the earliest commencement and the longest continuous time series datasets in the SEER database. The cancer database includes all diagnoses at these sites of each cancer type. Here, all types of skin cancer present in the database are included in the analysis. While it may be preferable to perform this analysis on individual skin cancer types with particular attention to those with greater known associations with UV exposure, this initial search for the presence of a global long term solar cycle component in skin cancer variation called for inclusivity for detection even if it is at the expense of eventual model fit.

### 2.2. Transforming the Data

For the analysis it is necessary to prepare the data and establish common unit time measures, in this case monthly observations. While several of the variables include measurements on shorter units of time, employing shorter units of measure becomes unnecessary when exploring long term trends, global scale changes, and events with great periods of latency. SN and TSI data sets have a time series representation with summarized monthly observations. To convert the SC case dataset to the same observational time scale we collapse cases into the count within each month.

**Figure 1:**
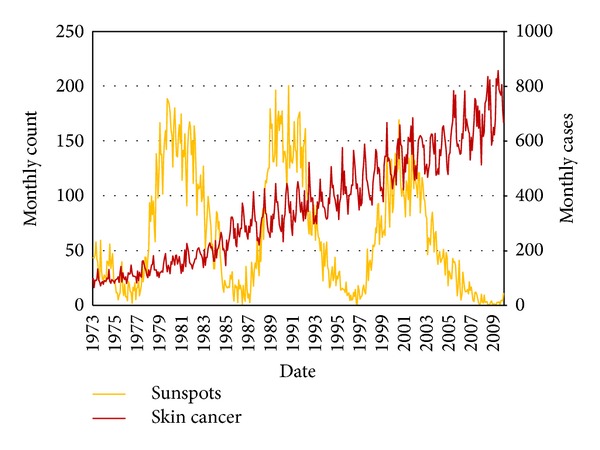
SN and SC monthly data.

Upon initial inspection, skin cancer case data exhibits a nonconstant variance among the observations as well as a growth rate which one would expect from changing population statistics across time (Figure 1). The natural logarithm transforms the case data and helps to stabilize the variance. Furthermore, natural logs provide a convenient interpretation that will be utilized later in the study by transforming differences of observations away from count or log count toward measures on a percentage scale. The other advantage of particular benefit with time dependent observations is the comparability of measures that previously were unique to populations of a certain specified time period.

The long term growth rate mentioned appears to have two distinct periods with different rates occurring before and after 1984 approximately. The possible reasons for this change are numerous and worth investigation but unexplored in the course of this research. For the purpose of this analysis, the SC data used spans 1984 through 2009, a period of relatively stable and consistent growth. In time series analysis the linear trend must be removed from the natural logarithm transformed SC cases. The linear trend in log scale corresponds to an exponential growth in the original case data. After trend removal, the remaining deviations from the trend comprise our dataset for continued analysis.

The absence of any sizable long term linear trends throughout our time period of interest for SN and our other datasets representing solar activity makes a similar process of trend analysis and removal unnecessary. In fact, solar activity does exhibit even longer term patterns of fluctuation, patterns across centuries, much greater than the 11-year period of interest [17]. However, the limits of the time frame of this study make these even longer term fluctuations appear relatively trendless given the shorter window.

### 2.3. Spectral Analysis

**Figure 2:**
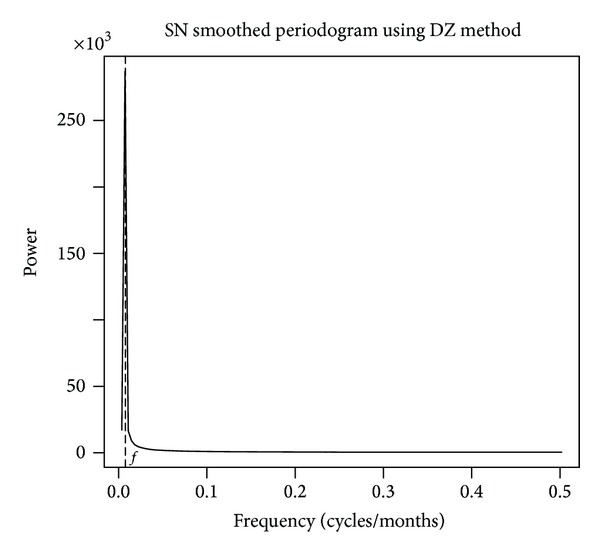
SN spectra resulting from application of KZP algorithm with parameters *m* = 443 and *k* = 1 and DiRienzo-Zurbenko (DZ) smoothing parameter = 1%, with frequency *f* corresponding to 11 years.

Most visible in the frequency domain, different time scales are likely rooted in different physical processes and thus arise from different causes. Our datasets viewed in a time domain appear as a compilation of the various influential time scales. Each dataset exhibits several strong features indicative of their respective time scales. The solar data most prominently exhibit a cyclic pattern with an approximate 11-year period. This is the solar cycle referenced. A smoothed Kolmogorov-Zurbenko periodogram displays a spike at the frequency, *f*, corresponding to 11 years (Figure 2). Adaptively smoothing the noisy raw periodogram using the DiRienzo-Zurbenko smoothing method allows the window size to vary with the underlying spectral density [18].

**Figure 3:**
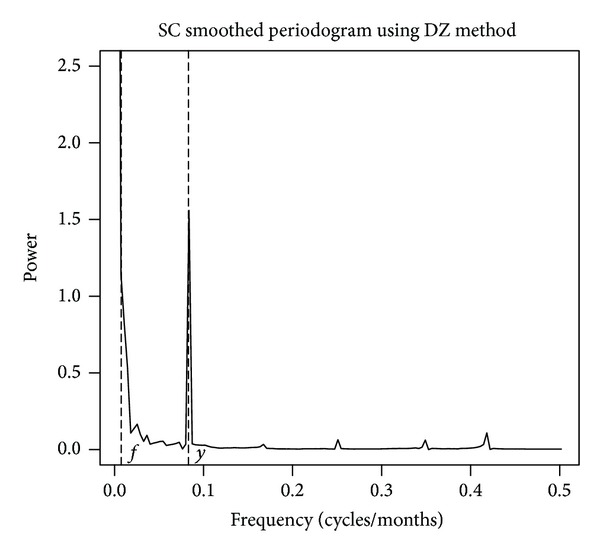
Ln(SC) spectra resulting from application of KZP algorithm with parameters *m* = 443 and *k* = 1 and DiRienzo-Zurbenko (DZ) smoothing parameter = 0.002%, with frequencies *f* corresponding to 11 years and *y* corresponding to 1 year.

Viewing SC in the time domain exhibits different characteristics. The first characteristic was the visible upward trend across time discussed previously. The second is a cyclic pattern that appears to repeat with an approximate one-year period. A corresponding DZ smoothed periodogram has a peak near 0 corresponding to the trend and a spike at a frequency corresponding to 1 year (Figure 3). These time scales represent the majority of the variation in skin cancer cases. Less apparent is the presence of a cycle at the frequency corresponding to the 11-year time scale.

### 2.4. Separation of Time Scales with KZ Filters

**Figure 4:**
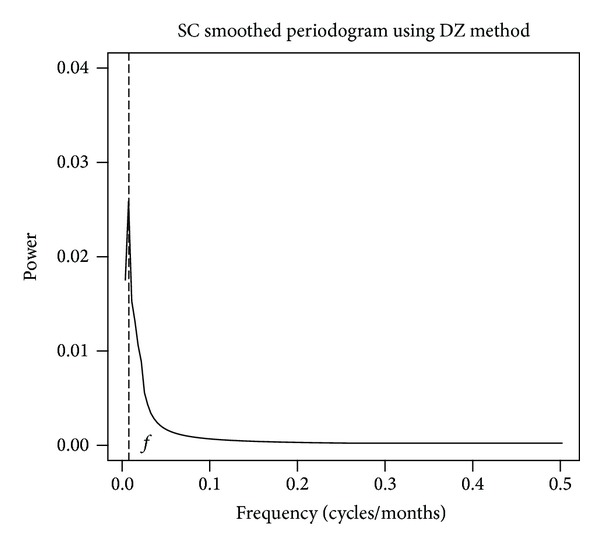
KZ filtered Ln(SC) spectra resulting from application of KZP algorithm with parameters *m* = 443 and *k* = 1 and DiRienzo-Zurbenko (DZ) smoothing parameter = 0.4%, with frequency *f* corresponding to 11 years.

Due to the strength of signals present throughout other time scales, a given time scale of relatively less signal strength may be obscured. In order to investigate a particular time scale it is necessary to separate and remove those that are interfering. Kolmogorov-Zurbenko filters are low pass filters characterized by two parameters [19]. With notation KZ(*k*, *m*) they are *k* iterations of a standard moving average filter of *m* points defining the moving average filter window. With interest in separating and filtering time scales, they are well suited in this study [20]. The presence of strong yearly cycles in some of our datasets, as well as known naturally occurring annual processes associated with solar activity, makes a twelve-month window the natural choice. Even numbered moving averages do not preserve the observational center point. To accommodate, this study uses a modified 13-month moving average, preserving the center point of the filter and weighting the first and last month of the window by one half. The first moving average iteration removes most signals with a period equal to or shorter than one year in each dataset. After the second moving average iteration, the KZ(13,2) filters have effectively removed all significant variations in this short term time scale, while leaving longer time scales unaffected [21]. This eliminates the strong random noise and seasonal components. With the trend previously removed, what remains in the smoothed periodogram is the long term (>1 year) time scale with the corresponding frequency associated with the 11 years noted (Figure 4).

### 2.5. Crosscorrelations

The data sets are then crosscorrelated to better understand the relationship between them. Note that for this study each pairwise crosscorrelation between two datasets only utilizes observed points beginning with the latest commencement of any dataset timespan and likewise ending at the first cessation of any dataset timespan. To account for possible latencies, or lags, in any possible causal relationship, data points from one variable are paired with opposing data points counted backward in time by *t* steps, or a lag *t*, prior to calculating the correlation. Crosscorrelations are calculated first with lag *t* = 0, or no latency, and then for all integers lags up to some reasonably large value. There are indications of several optimal lags for maximizing correlations. Two sinusoidal signals with a peak correlation occurring at lag *t* will also naturally have peak correlations when the lag is an integer multiple of the signal period away from *t*. The choice of which lag is most appropriate can be guided by several factors, in this case maximization, but bounded within the lower limit of disease onset and the upper limit of human lifespan. A reasonable range here might span from 0 to 70 years.

### 2.6. Regression Analysis

After crosscorrelations are calculated for all possible latencies, the latencies associated with peak correlations are selected and used to perform regression analysis between the variables. Regression analysis, in this case simple linear regression, provides a good fit and allows us to characterize the relationship and see how one variable is associated with the movement in another. Finally, the coefficient of explanation, *R* ^2^, created by squaring the correlation coefficient at the chosen latency provides a measure to determine the fraction of variance of one variable explained by another. It should be noted that correlation is typically used in least squares estimation where observations should be independent. In time series analysis, particularly with the application of moving average filters, consecutive points are highly correlated. The use of correlation and subsequently the coefficient of explanation, *R* ^2^, in this analysis is a measure of the quality of fit. It uses identical calculations as the standard statistical correlation with an interpretation of the percentage of total variance explained by the fit of one variable to another.

## 3. Results

### 3.1. SN, OTSI, and GTSI

**Figure 5:**
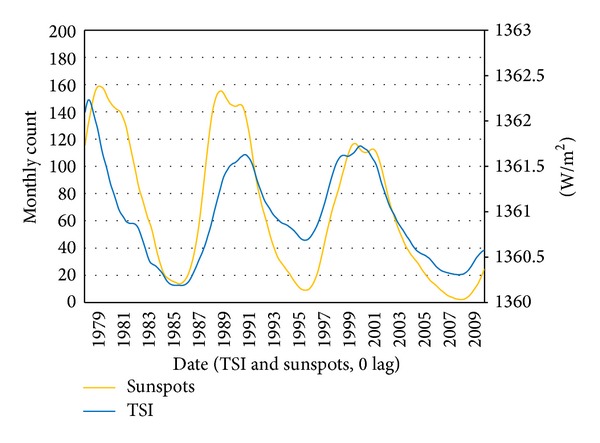
SN and OTSI long term (>1 yr) time scale components.

After KZ(13,2) filters are applied to SN, OTSI, and GTSI, crosscorrelation between paired datasets confirms the strong correlations between the long term variation of each of these variables. The peak value in crosscorrelation between SN and OTSI is *r* = 0.80 (Figure 5). This peak occurs at zero latency, and each of these pairings strongly exhibits the approximate 11-year solar cycle in crosscorrelations.

**Figure 6:**
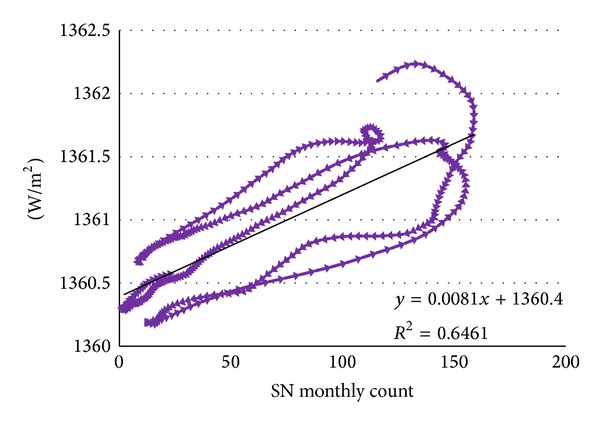
OTSI plot versus SN with 0 latencies.

Pairing OTSI and SN at 0 latency enables characterization of the relationship between these variables. Fitting a linear regression model produces a slope coefficient of 0.0081 (Figure 6).

The slope coefficient indicates an increase in 0.0081 W/m^2^ associated with each additional monthly sunspot count, or, more befitting the range, 0.81 W/m^2^ per 100 SN. This value will enable extension of the analysis from SN to TSI regardless of the short observational history.

### 3.2. Skin Cancer

With SC it was necessary to transform the data prior to crosscorrelation. First was the previously described natural logarithm transformation. Second was the removal of an upward trend. The construction of a linear trend using least squares estimation resulted in a slope coefficient of 0.0034. Given the natural log scale, 0.0034 corresponds to approximately 4.2% growth per year. Crosscorrelations between SC cases reach maximum correlations *r* = 0.51 with GTSI, 0.58 with OTSI, and 0.63 with SN. Here GTSI and OTSI datasets have insufficient history to investigate latency with SC beyond a small number of years. While these datasets are too limited to fully examine the potential latency of skin cancer, their cyclic nature and strong correlation coefficient with both SN and SC are still supportive of the results observed between SC and SN. SN is the only solar cycle variable with a sufficient history to crosscorrelate with SC approaching the mean individual lifespan, a natural upper limit for cancer latency.

**Figure 7:**
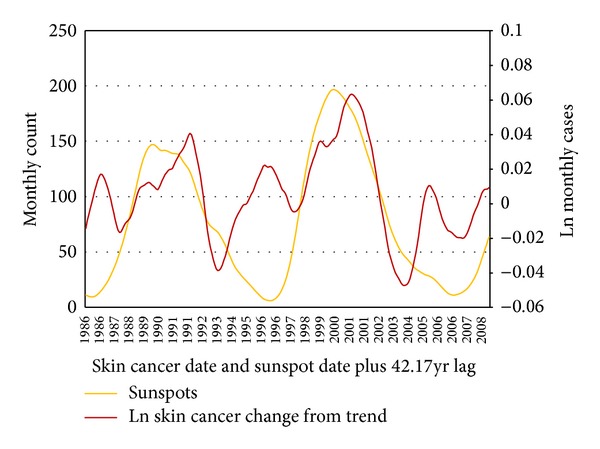
SN and Ln SC long term (>1 yr) time scale components.

SC crosscorrelations with SN peak at candidate latencies of 10.0, 19.9, 31.8, 42.2, 52.3, and 62.5 years, and so forth. These peak crosscorrelations range from a minimum of 0.34 occurring at 31.8 years to a maximum correlation of 0.68 at 42.2 years (Figure 7).

These correlations correspond to coefficients of explanation, ranging from *R* ^2^ = 0.12 to 0.39. Evidence visible in Figure 7 and derived from the coefficient indicates that there are other complex long term influences present at other long term frequencies. Investigation of these lesser effects at other frequencies is worthwhile particularly if any future analysis attempts to completely decompose the SC time series. However, given that the 11-year frequency has the strongest long term effect outside of the SC trend and seasonal component and given the importance of this particular frequency in investigating the solar influence on SC, we limit the analysis to this frequency of interest. The time series analysis of this study does not provide tools to indicate preference or likelihood of one candidate latency over another. However, given that the 42.2-year latency period is consistent with evidence of the delay between initiation and detection of skin cancer and that crosscorrelation maximizes at this latency, investigating these latencies with particular attention to that at 42.2 years is reasonable.

**Figure 8:**
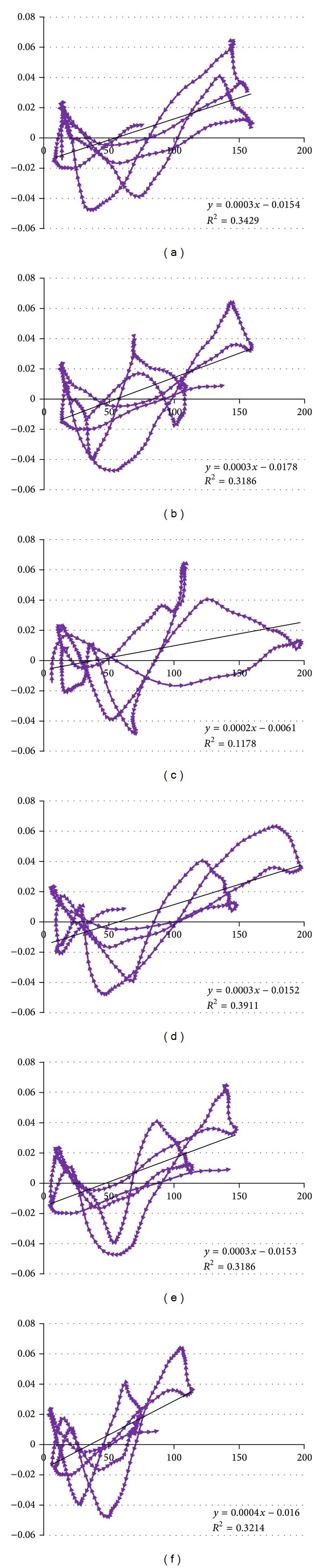
Deviation of LN skin cancer monthly cases versus SN monthly count with the (a) 10.0, (b) 19.9, (c) 31.8, (d) 42.2, (e) 52.3, and (f) 62.5 years of latency.

With the peaks in crosscorrelation at these candidate years, we plot our transformed SC dataset, best summarized as the deviations from the natural log SC trend, against SN data that is lagged by those respective latencies (Figure 8). These plots help visualize the strong correlation at each latency. Each plot notes the coefficient of explanation indicating the quality of fit as well as the slope coefficient of linear regression. For example, in reference to the plot for the 42.2-year latency recall using the calculated crosscorrelation of 0.63 to compute a coefficient of explanation, or *R* ^2^, of 0.3911. Fitting a linear regression model to the lagged data produces a slope coefficient of 0.0003 (per one additional sunspot monthly count) or, more appropriately befitting the scale, 0.03 per an increase in 100 SN. This corresponds to a 3.05% increase in skin cancers per 100 SN.

## 4. Discussion

Evidence suggests there is a relatively small but distinct solar cycle effect on long term SC case variation. The relative influences from other time scales, such as the long term trend and seasonal component, cloak this long term solar cycle effect. Kolmogorov-Zurbenko filters provide an effective tool to separate and screen interfering time scales. Identification of this effect is possible by the separation from the influence of other uncorrelated time scales. Although this effect accounts for only a small percentage of the total variation in skin cancer incidence the benefit of investigating this particular frequency is not available using other time scales. Here the solar cycle fingerprint enables an analysis of the coefficient of influence with this singularly identifiable source which does not exist at other time scales. Crosscorrelation at different latencies accounts for the unknown delay between risk exposure and cancer detection. The latency of peak crosscorrelation is used to determine the magnitude of long term effects and characterize the relationship between variables. Once identified, the coefficient of influence between changes during the solar cycle and SC can be applied to actual observed changes in solar intensity in other time scales even when the underlying source is indeterminate. It should be noted that the ecological design of this study, while providing a risk modifying analysis of the health effect, has both advantages and disadvantages. It is well suited for the analysis of data grouped in this case both geographically and across time. This comes at the expense of generalized conclusions for the population at large that may not apply individually. In this case, this does not hinder an attempt to identify the global scale, long term component of skin cancer variation.

TSI is a natural choice as a representative variable of the solar cycle effect on skin cancer. The known risk of ultraviolet light exposure on skin cancer is a compelling argument in favor of its use. Unfortunately at this time, without additional years of observation, the need for a sufficient history to both detect an 11-year cycle and account for a multidecade latency makes TSI or any specific segment of the electromagnetic spectrum such as ultraviolet light unsuitable. These more accurate TSI records, though of limited research potential here, are however supportive of the analysis that can be performed with SN. Although orbital TSI has the shortest history and ground based TSI suffers from regional influences limiting its usefulness to study global effects, both produced results similar to and compatible with that obtained using SN. Crosscorrelations with SC in the long term time scale component gave evidence of the presence of the solar cycle effect. The extension of the crosscorrelation analysis requires a far lengthier history to investigate reasonable cancer latencies. In the future, with several additional years of data, extending this analysis using TSI measures may produce interesting and even more definitive results.

With SN as the only tenable solar cycle variable with sufficient history, the study proceeds by removing the linear trend from natural logarithm transformed SC case data. With a linear regression coefficient of 0.0034 on the log scale, this indicates that the rate of growth in skin cancer cases for several decades is approximately 4.2% per year. This outpaces the approximate 1% population growth in the United States during a similar period. Clearly, population growth can not alone account for the growth in skin cancer diagnoses, an interesting result and one worthy of continued investigation. Also, prior to 1984, SC data suggests a steady though lower growth rate than that after 1984. Future analysis could be extended to include data from this earlier period following a more detailed analysis of the reasons behind the abrupt rate change and properly accommodate for this feature.

Crosscorrelations between SN and SC displayed the cyclic pattern with an approximate 11-year synchronicity when plotted at different latencies, further supporting the presence of a solar cycle component. These crosscorrelations attain a peak value of *r* = 0.68 at the 42.2-year latency. The square of this correlation produces the coefficient of explanation, *R* ^2^ = 0.39. Recalling that the transformed SC was on a natural log scale and had a linear trend removed and that both SC and SN data had the KZ filter applied to only retain the long term component, we can properly interpret the coefficient for this latency. A suitable interpretation is that 39% of the long term (>1 year) variation in skin cancer (in log scale) deviation from the trend can be explained by the variation in SN that occurred 42.2 years prior.

When plotting SC versus SN at the given latencies corresponding to each peak crosscorrelation, the association between the long term skin cancer and sunspot number datasets can be described by a linear relationship with a linear coefficient. These slope coefficients share very similar values near 0.0003, with only two exceptions creating a range from 0.0002 to 0.0004. While the time series tools do not indicate a preference for one particular latency, the same linear coefficients produced do not necessitate selection to provide an interpretation of the relationship between SC and SN. Given the log skin cancer scale the coefficient of linear regression has a clear interpretation by differencing the natural logarithm transformed values resulting in a percentage scale. A typical solar cycle can decrease to a near zero sunspot count in a given month and can peak at 150, 200, or even 250 sunspots during solar maximum [22]. Using the derived linear coefficient of 0.0003 and these typical solar cycle amplitudes, they represent associated increases in skin cancer cases of 4.6, 6.2, and 7.8 percent, respectively. Therefore, choosing the maximal correlation at the 42.2-year latency, a typical solar maximum with 200 monthly sunspots is associated with 6.2% more monthly SC cases 42.2 years later, when compared to a solar minimum. The strong correlation and near synchronous relationship between SN and TSI allow the extension of this result. During a typical solar cycle, orbital TSI indicates that irradiation varied by only 1.6 additional W/m^2^, an increase in TSI of only 0.1% [12]. TSI data limitations prevent direct crosscorrelations at long latencies but the strong correlation between OTSI and SN makes it reasonable to similarly model the effect of small changes in TSI on SC cases. Thus, 1.6 additional W/m^2^ is associated with the 6.2% increase in monthly SC cases, 42.2 years following a solar maximum, as compared to a solar minimum.

An immediate extension of this analysis in future research is the application of the same methods to individual skin cancer types with both known sun exposure risk factors and those that have conflicting evidence as to the effect of sun exposure. In order to reveal the existence of a small and obscured solar cycle effect this study relied upon including all skin cancers for sufficient history and data records. This comes at the expense of including possibly uncorrelated cancer types, diminishing signal strength and reducing model fit. Provided that sufficient data resources are available, future analyses using this method applied to investigate particular skin cancers may be more illuminating and result in more refined models.

Knowing that associated skin cancer risk increases with increased solar activity during solar maximums and that this occurs in a well-known, predictable, cyclic pattern, there is opportunity to more effectively target education and prevention campaigns aimed at reducing skin cancer prevalence. The methods outlined in this analysis are equally applicable to similar research where the detection of a signal within a particular time scale is obscured by relatively stronger signals from different time scales, or by destructive noise. This research could be extended to the relationship between the solar cycle and other diseases that may have a long term hidden effect, or to other risk factors of disease.

Within the scope of this research project, the data was limited to records obtained within the United States. With additional datasets, particularly those outside of the US, extending this research would better clarify results to more accurately determine true global long term effects of the solar cycle. The Kolmogorov-Zurbenko filter has previously been extended and formalized in several useful applications including a spatial filter. With additional existing data elements it is possible to extend this research to include spatial data from the cancer database. Rather than pooling the data for a global effect it would then be possible to determine regional effects and develop regional models. This could first be performed by banding latitudes to account for the effect of latitude on irradiation intensity. Secondly, the analysis could be refined to individual locations accounting for local variation in meteorology and geography.

These are just a few of the possible extensions and applications of the research methods and results outlined in this study. Modeling and forecasting are only likely to improve with improved data, additional years of observations, and the inclusion of more accurate representative solar radiation variables as they become available, highlighting the need for continued TSI data collection. This study illustrates the importance of investigating long term effects that may be hidden by other time scales or noise but that significantly contribute to the understanding of disease risk and prevention.

## References

[1] Rogers HW, Weinstock MA, Harris AR (2010). Incidence estimate of nonmelanoma skin cancer in the United States, 2006. *Archives of Dermatology*.

[2] Pfahlberg A, Kölmel K-F, Gefeller O (2001). Timing of excessive ultraviolet radiation and melanoma: epidemiology does not support the existence of a critical period of high susceptibility to solar ultraviolet radiation-induced melanoma. *British Journal of Dermatology*.

[3] Lew RA, Sober AJ, Cook N, Marvell R, Fitzpatrick TB (1983). Sun exposure habits in patients with cutaneous melanoma: a case control study. *The Journal of Dermatologic Surgery and Oncology*.

[4] Koh HK, Geller AC, Miller DR, Grossbart TA, Lew RA (1996). Prevention and early detection strategies for melanoma and skin cancer: Current status. *Archives of Dermatology*.

[5] Parkin DM, Mesher D, Sasieni P (2011). Cancers attributable to solar (ultraviolet) radiation exposure in the UK in 2010. *British Journal of Cancer*.

[6] Keller AK, Uter W, Pfahlberg AB, Radespiel-Tröger M, Gefeller O (2013). Seasonality of cutaneous melanoma diagnoses: a comprehensive comparison of results in Bavaria and Northern Ireland. *Melanoma Research*.

[7] Wang Y-M, Lean JL, Sheeley NR (2005). Modeling the Sun's magnetic field and irradiance since 1713. *The Astrophysical Journal Letters*.

[8] Lean JL, Rind DH (2009). How will Earth's surface temperature change in future decades?. *Geophysical Research Letters*.

[9] Foukal P, Lean J (1988). Measurements of facular photometric contrast. *The Astrophysical Journal*.

[10] Sheeley NR (1991). Polar faculae: 1906–1990. *Astrophysical Journal Letters*.

[11] Lean J (2000). Evolution of the Sun's spectral irradiance since the Maunder Minimum. *Geophysical Research Letters*.

[12] Fröhlich C, Lean J (2004). Solar radiative output and its variability: evidence and mechanisms. *Astronomy and Astrophysics Review*.

[13] Nadler DL, Zurbenko IG (2013). Developing a weibull model extension to estimate cancer latency. *ISRN Epidemiology*.

[14] Tsakiri K, Zurbenko I (2011). Effect of noise in principal component analysis. *Journal of Statistics and Mathematics*.

[15] Willson RC, Mordvinov AV (2003). Secular total solar irradiance trend during solar cycles 21–23. *Geophysical Research Letters*.

[16] Hempelmann A, Weber W (2012). Correlation between the sunspot number, the total solar irradiance, and the terrestrial insolation. *Solar Physics*.

[17] Lean J, Beer J, Bradley R (1995). Reconstruction of solar irradiance since 1610: implications for climate change. *Geophysical Research Letters*.

[18] DiRienzo AG, Zurbenko IG (1999). Semi-adaptive nonparametric spectral estimation. *Journal of Computational and Graphical Statistics*.

[19] Zurbenko IG (1986). *The Spectral Analysis of Time Series*.

[20] Zurbenko IG, Cyr DD (2011). Climate fluctuations in time and space. *Climate Research*.

[21] Close B, Zurbenko I Kolmogorov-Zurbenko adaptive filters. (Version 3). http://cran.r-project.org/web/packages/kza/index.html.

[22] Solanki SK, Fligge M (1999). A reconstruction of total solar irradiance since 1700. *Geophysical Research Letters*.

